# Smoothed millennial-scale palaeoclimatic reference data as unconventional comparison targets: Application to European loess records

**DOI:** 10.1038/s41598-020-61528-8

**Published:** 2020-03-25

**Authors:** Christian Zeeden, Igor Obreht, Daniel Veres, Stefanie Kaboth-Bahr, Jan Hošek, Slobodan B. Marković, Janina Bösken, Frank Lehmkuhl, Christian Rolf, Ulrich Hambach

**Affiliations:** 1 LIAG, Leibniz Institute for Applied Geophysics, Hannover, Germany; 2 IMCCE, Observatoire de Paris, PSL Research University, CNRS, Sorbonne Universités, UPMC Univ. Paris 06, Univ. Lille, Paris, France; 3 Organic Geochemistry Group, MARUM-Center for Marine Environmental Sciences and Department of Geosciences, University of Bremen, Bremen, Germany; 4 Romanian Academy, Institute of Speleology, Cluj-Napoca, Romania; 5 Institute of Earth Sciences, Ruprecht-Karls-Universität Heidelberg, Heidelberg, Germany; 6 Institut für Geowissenschaften, Universität Potsdam, Potsdam, Germany; 7 Czech Geological Survey, Prague, Czech Republic; 8 Center for Theoretical Study, Charles University and the Academy of Sciences, Prague, Czech Republic; 9 Chair of Physical Geography, Faculty of Sciences, University of Novi Sad, Novi Sad, Serbia; 10 Department of Geography, RWTH Aachen University, Aachen, Germany; 11 BayCEER & Chair of Geomorphology, University of Bayreuth, Bayreuth, Germany

**Keywords:** Palaeoclimate, Stratigraphy, Sedimentology

## Abstract

Millennial-scale palaeoclimate variability has been documented in various terrestrial and marine palaeoclimate proxy records throughout the Northern Hemisphere for the last glacial cycle. Its clear expression and rapid shifts between different states of climate (Greenland Interstadials and Stadials) represents a correlation tool beyond the resolution of e.g. luminescence dating, especially relevant for terrestrial deposits. Usually, comparison of terrestrial proxy datasets and the Greenland ice cores indicates a complex expression of millennial-scale climate variability as recorded in terrestrial geoarchives including loess. Loess is the most widespread terrestrial geoarchive of the Quaternary and especially widespread over Eurasia. However, loess often records a smoothed representation of millennial-scale variability without all fidelity when compared to the Greenland data, this being a relevant limiting feature in integrating loess with other palaeoclimate records. To better understand the loess proxy-response to millennial-scale climate variability, we simulate a proxy signal smoothing by natural processes through application of low-pass filters of δ^18^O data from Greenland, a high-resolution palaeoclimate reference record, alongside speleothem isotope records from the Black Sea-Mediterranean region. We show that low-pass filters represent rather simple models for better constraining the expression of millennial-scale climate variability in low sedimentation environments, and in sediments where proxy-response signals are most likely affected by natural smoothing (by e.g. bioturbation). Interestingly, smoothed datasets from Greenland and the Black Sea-Mediterranean region are most similar in the last ~15 ka and between ~50–30 ka. Between ~30–15 ka, roughly corresponding to the Last Glacial Maximum and the deglaciation, the records show dissimilarities, challenging the construction of robust correlative time-scales in this age range. From our analysis it becomes apparent that patterns of palaeoclimate signals in loess-palaeosol sequences often might be better explained by smoothed Greenland reference data than the original high-resolution Greenland dataset, or other reference data. This opens the possibility to better assess the temporal resolution and palaeoclimate potential of loess-palaeosol sequences in recording supra-regional climate patterns, as well as to securely integrate loess with other chronologically better-resolved palaeoclimate records.

## Introduction

### Millennial-scale climate variability

Millennial-scale climate variability, namely the Greenland Interstadials (GI) and Stadials (GS) is documented in ice-core, marine and speleothem records from the Northern Hemisphere during the last glacial cycle. These rapid palaeoclimate events (GI and GS) induced changes in the terrestrial environments on land e.g.^1–10^. However, for most palaeoclimate records the synchronicity of change is hard to establish due to dating uncertainties and different sensitivity to past climate forcing^11–13^. Increasing the analytical resolution does not necessarily solve these issues, and a recent study^14^ argues that “discrepancies remain in the temporal development of rapid climate change for specific events”.

### Low-pass filters and their properties

Low-pass filters are a way of omitting high-frequency oscillations of a particular dataset and focusing on the low-frequency (long wavelength) part of the signal. Before filtering, datasets are linearly interpolated. The interpolation resolution will determine the number of data points, and the process can change the auto-correlation of datasets. It needs to be mentioned that filters, as also other time series analyses techniques, commonly have edge effects e.g.^15,16^ at the beginning and end of datasets. In practice, this means that filters may not reliably represent data at the beginning and end of datasets. Therefore, the patterns of filters around these intervals should not be interpreted in detail.

**Figure 1:**
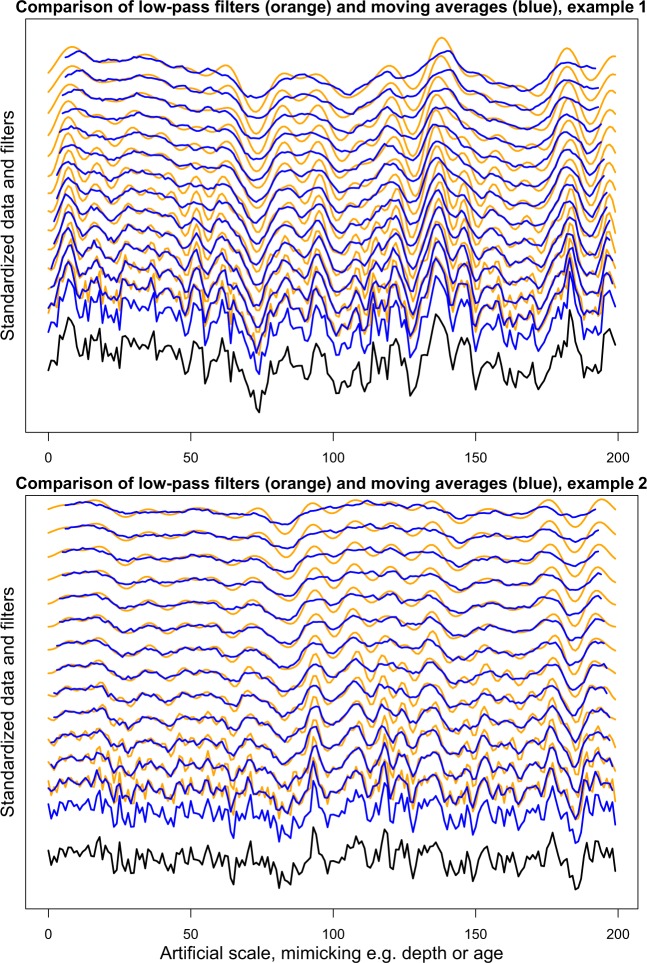
Comparison of low-pass filters (orange) and moving averages (blue) on two artificial datasets. An example of a highly auto-correlated signal (top; auto-correlation coefficient 0.9), and a less auto-correlated and ‘more noisy’ signal (bottom; auto-correlation coefficient 0.5). Note that especially for the second (noisier) example filters focus more on longer-term trends and are less affected by high-frequency signals, which may represent noise. From bottom to top the moving average window width and cut-off frequency increases from 1 to 14 scale units.

Smoothing of noisy data is a basic principle of data- and signal processing allowing for the extraction of longer-term patterns and avoidance of high-frequency fluctuations, which may represent noise^17^. While the mean of moving windows smooth data directly, filters focus on specific frequency ranges. Low-pass filters extract the long-term variability of a dataset and exclude the high-frequency content. Band pass filters are widely used to extract information about specific frequency components especially of orbital climate forcing e.g.^18–22^. Low-pass filters have been used to focus on the low frequency components of solar^23^, millennial^24^ and orbital e.g.^25–28^ signals with implications in defining past climate variability. Although the concept of low-pass filters is similar to a moving average (see Fig. 1 for a comparison of two examples), filters have the advantage that the frequency response can be constrained by adjusting the roll-off rate, a parameter for the width of the frequency response of filters. This feature makes filters a powerful tool, especially when flexibility in their design (cut-off frequencies and roll off rate) is provided. This is the case for Taner filters as implemented in the ‘astrochron’ R package^29–31^. Here, Taner low-pass filters are used to investigate smoothed signals in ice cores and speleothems, as approximation/representation/simulation for smoothing due to e.g. loessification (post-depositional alteration including weak pedogenesis) combined with low sedimentation rates e.g.^32,33^ for loess records.

### Loess proxy records and their chronologies on millennial time scales

Loess records form one of the most widespread terrestrial palaeoclimate archives in the mid latitudes of Northern Hemisphere landmasses e.g.^34–37^. Limitations induced by proxy-response time, thresholds and tipping points often result in smoothing of the primary climate signal by syn- and post-sedimentary bioturbation and/or chemical weathering. Therefore, a uniform understanding of loess palaeoclimate data in the light of millennial-scale past climate variability is not always well constrained, albeit patterns of millennial-scale climate variability can be identified in many records e.g.^4,12,13,38,39^. It is important to note that different processes can be expected to act on different depth- and time scales. Bioturbation can range from mm to decimetre-scale burrows. In addition, dissolution in the underground including sinkholes can appear and disturb the stratigraphy. The depth and intensity of soil formation will depend on amongst others the vegetation type. Therefore, our approach of smoothing a record needs to be considered a simplification trying to sum all smoothing mechanisms, which in reality act on different depth- and time scales.

As millennial-scale climate variability has been documented in various Eurasian records e.g.^1,4,5,10,39–48^, it can be expected that its imprint would be discernible also in low-resolution loess records.

### Chronologies for loess geoarchives: Dating and correlation

Constraining reliable chronologies for loess records beyond radiocarbon dating is especially challenging because of considerable uncertainty of luminescence dating techniques, the main methods applicable to dating the emplacement time of mineral-particles forming loess e.g.^49–52^. Their precision and accuracy is often unable to provide the dating resolution needed for unambiguous assignment of palaeoclimatic signals to reference datasets such as independently dated ice cores^53–56^ or speleothem data e.g.^1,6,9^. This commonly leaves inferred correlation of supposedly (quasi)identical past climate events as the only method to establish high-resolution chronologies for loess. However, several loess proxy-data show less high-frequency oscillations on millennial time scales than high-resolution reference records e.g.^57–61^, challenging detailed pattern-matching attempts. To counter this issue at least partly, we suggest to smooth (technically: low-pass filter) high-resolution reference data e.g.^1,54,62^ before attempting comparison and correlation of palaeoclimate events among loess records or between loess and other palaeoclimate archives. This is expected to facilitate the comparison of datasets with similar temporal resolution leading to a better understanding of proxy-response and palaeoclimate potential of such records.

Correlative age models relying on the identification of similar patterns in loess and well established reference records could provide more reliable age-depth relationships especially when verified by independently dated tie-points such as tephra layers^3,12,63–65^. Correlative age models have proven crucial for time scale development especially on orbital and millennial time scales e.g.^66–68^. Prominently orbital tuning, magnetostratigraphy and also isotope- and event stratigraphy are based on visual or signal correlation, though often supported by an integrated stratigraphic approach using several independent dating techniques^69–73^. Although correlative age models may be regarded as reliable geochronometric methods^71,74,75^, correlation may impose artificial patterns in data series^28,76–80^. Further, such time scales may be hardly reproducible and are often based on individual choice and experience. Several methods have been developed to overcome or limit ambiguities and allow for a more quantitative assessment of correlations, age models and their significance^28,29,77,80–87^. It may be regarded acceptable that correlation is most reliable when supplemented and supported by independent age control. Correlative age models and comparisons have been widely applied to loess research e.g.^4,39,88–96^, and many time scales older than the last glacial cycle are at least partly based on correlation to other geoarchives, usually marine or ice cores^54,97,98^ or orbital correlation targets^99^. As demonstrated for radiometric time scales^100^, also correlative time scales can be expected to provide unreliable constraints where no clear data patterns can be traced between records, or no tie points can be set without ambiguity. Especially because even large numbers of radiometric dates can hardly prove synchrony of events between records, we regard correlation besides dating of vital importance for improving loess palaeoenvironmental reconstructions.

## Results

**Figure 2:**
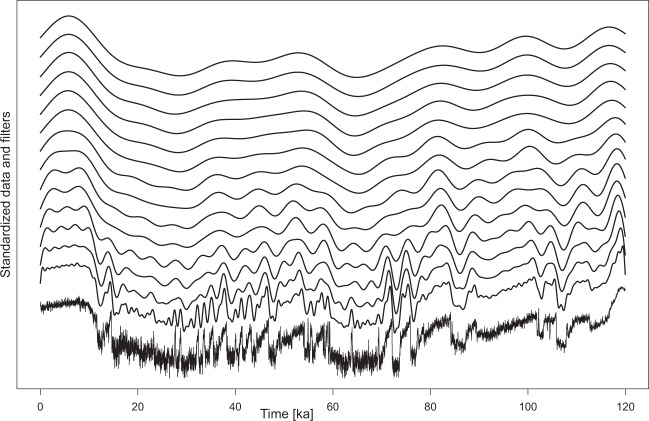
Low-pass filters with cut-off frequencies from 1–14 kyr (increasing from bottom to top) of the Greenland δ^18^O record, a record often used as correlation target for last glacial northern hemisphere climate evolution. All data were standardized, and low-pass filters are offset on the ordinate for plotting. Note the increasing smoothness with increasing cut-off frequency (bottom to top, from 1–14).

Results for the low-pass filtering of δ^18^O data from Greenland are performed in 1 kyr steps of the cut-off period from 1–15 ka (and thus the cut-off frequency of 1/2 to 1/15 [1/kyr]; Fig. 2). While the filtering with cut-off periods of 1–2 kyr indicates prominent millennial-scale climate variability, these patterns vanish with increasing cut-off frequency, and give way to patterns of several ka periods (cut-off period ~4–8 kyr) to orbital scale variability (cut-off period >10 kyr).

**Figure 3:**
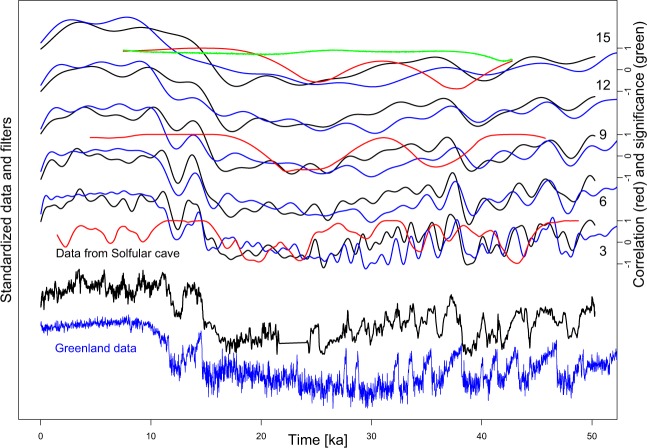
Comparison of two independently dated reference datasets for Europe and the Black Sea region: Greenland δ^18^O data and its smoothing by low-pass filtering (blue; cut-off period is indicated at the right from 4–12 a), and the Sofular cave δ^13^C data (black) data and low-pass filters with cut-off frequencies of 3, 6, 9, 12 and 15 kyr (see numbers at the right). Note general similarity, and dissimilarity especially between ~30 and 15 ka. Spearman rank correlation coefficients for the filters with cut-off frequencies at 3, 9, and 15 kyr are included (red), they each have axes on the right. The top panel include results of a correlation test (green; see chapters 3.1 and 5.3), correlation is significant where correlation of the dataset (red) is higher than for reference (green); this is the case from ~20 ka until present for the data filtered with a cut-off frequency of 15 kyr. Selected low-pass filters were used for correlation and significance to keep the figure clear.

The visual and quantified comparison of the Greenland δ^18^O data on the AICC2012 time scale^56^ and δ^13^C data from Sofular cave in northern Turkey^1^ suggests general similarity, but dissimilarity is also observable especially in the interval from ~30–15 ka (Fig. 3). The similarity of these datasets is especially high for the last ~15 ka and the time interval from ~40–30 ka. Correlation among those two reference records exceeds the significance at 95% confidence for cut-off frequencies of 15 ka (Fig. 3, top panel) for the last ~15 ka. In contrary, the time interval from ~25–15 ka shows little variability in the ice core record, a gap in the speleothem dataset, and generally pattern amplitudes are low (Fig. 3), resulting in no significant relationship.

**Figure 4:**
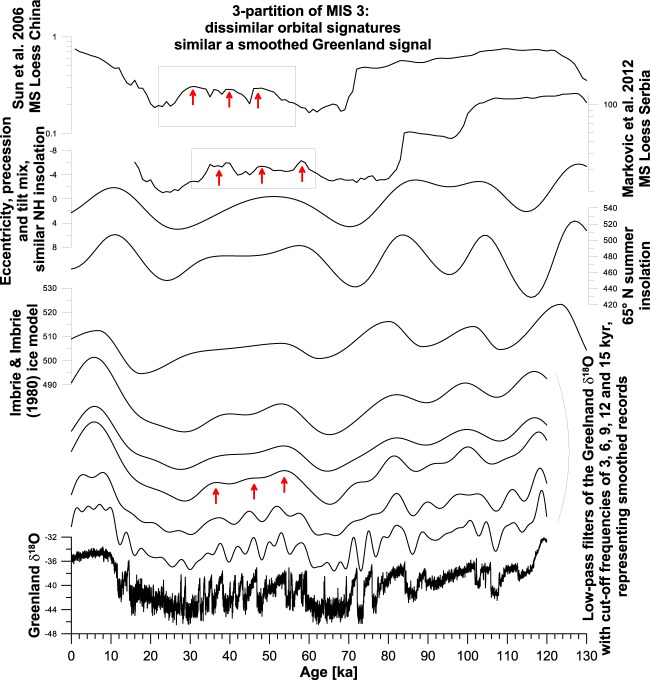
Original- and low-pass filters of Greenland δ^18^O data (bottom), compared with other established reference datasets from the last glacial cycle, and two datasets from loess. Reference datasets represent the ice model^124^, parametrized as for a marine benthic isotope stack^98^, summer insolation at 65 °N^99^, and an ETP mix of eccentricity, tilt (obliquity) and precession^99^. Loess datasets from the Carpathian Basin (Mošorin/Serbia)^113^ and China^114^ are displayed at the top. Please note a 3-division of enhanced magnetic susceptibility, which cannot be explained by classical correlation targets, but by a smoothed Greenland signal.

Next, magnetic susceptibility data from Chinese and European loess e.g.^61,101–105^ sometimes reveal for Marine Isotope Stage (MIS) 3 a soil formation phase mainly characterized by three prominent maxima in the magnetic susceptibility records (Fig. 4). These are difficult to explain by simple comparison to insolation and the Imbrie and Imbrie (1980) ice model (Fig. 4).

**Figure 5:**
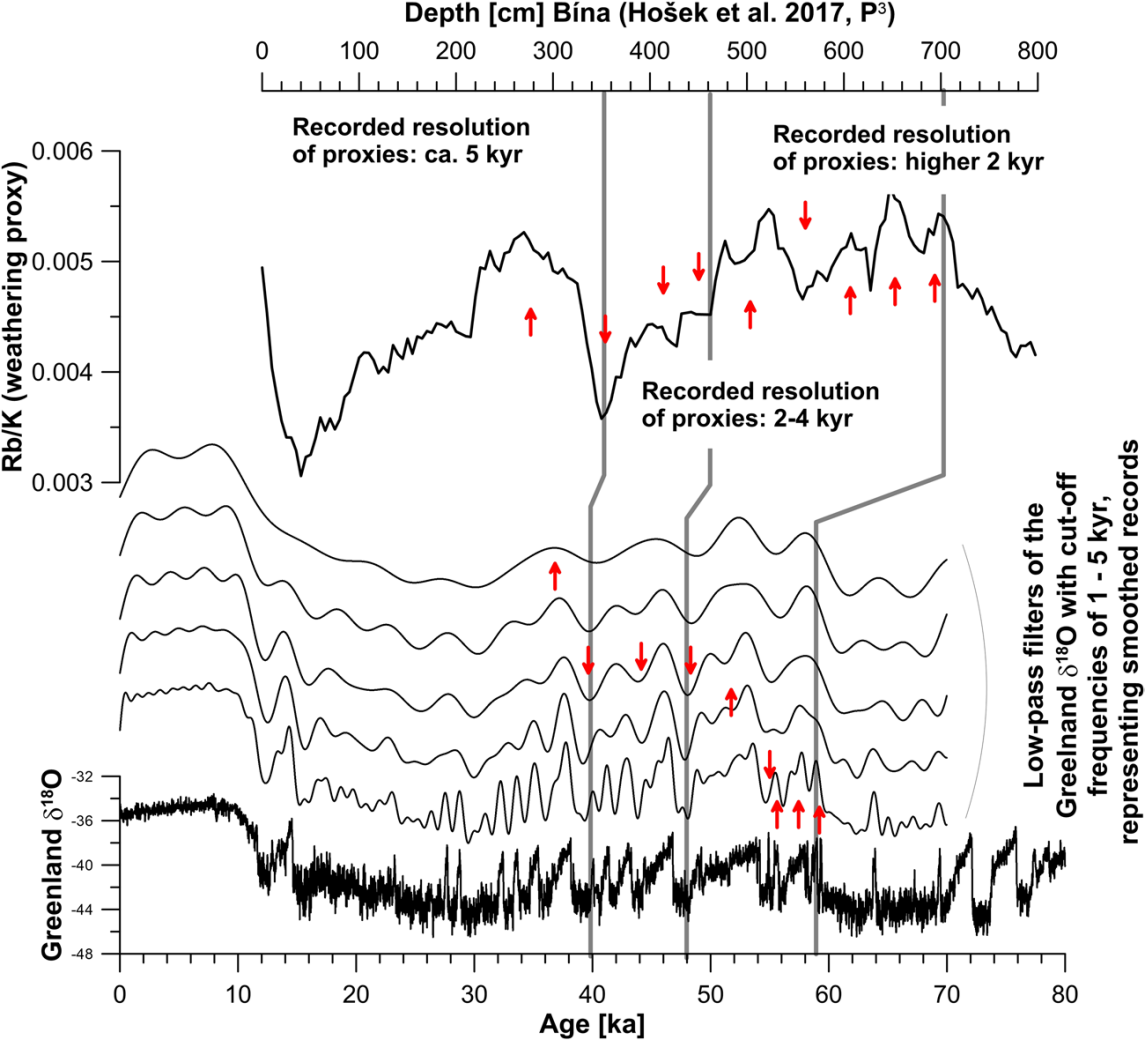
Comparison of the Rb/K signal from the Bíňa profile (luminescence dated)^106^ and low-pass filtered Greenland δ^18^O data. Note the different resolution recorded in the sequence. The higher resolution at the base is a consequence of its geomorphologic position in a depression, which was filled with sediment. Consequently, the top of the sequence has lower deposition rates, and also a lower recorded resolution. Vertical correlation lines after the original luminescence-supported correlation in^106^. Red arrows propose correlation of the signal to differently smoothed Greenland records.

In Fig. 5, the smoothed Greenland record is compared to the Rb/K ratio, a loess weathering proxy from the Bíňa section in the Czech Republic^106^. The comparison shows that some intervals are highly smoothed in the proxy dataset (especially the part younger ca. 40 ka), and that especially the time interval around 60 ka is expressed very well in the proxy record. The available time scale, based on a combination of luminescence dating and correlation to Greenland δ^18^O data^106^, may be refined by comparison and correlation to a smoothed Greenland signal. In particular, when compared to a suite of smoothed correlation targets, one can assess which of these resembles the variability in the proxy dataset best.

**Figure 6:**
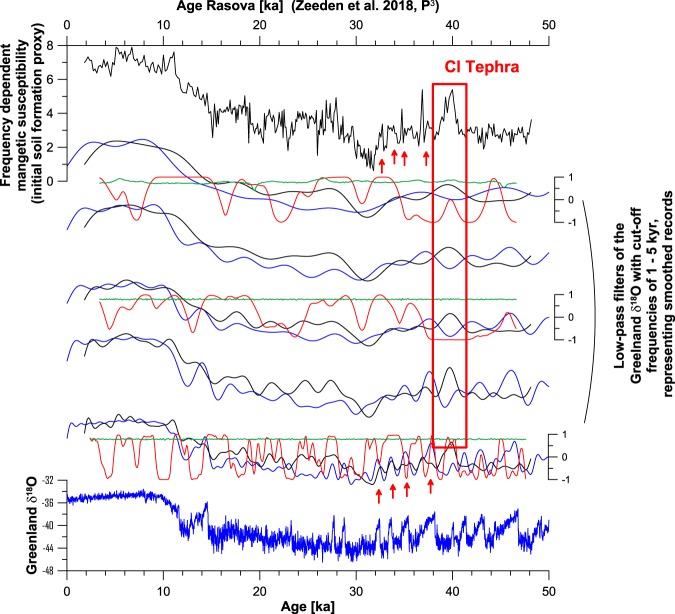
Comparison of Greenland δ^18^O data and its smoothing by low-pass filters (blue), with data of the frequency dependent magnetic susceptibility from Rasova at the Lower Danube^12^ (black). Correlation is quantified via the Spearman rank correlation to allow for some nonlinearity, and is included for three low-pass filters s in red. This selection is made to keep the figure clear. Significance is evaluated as well, and correlation for phase-randomized surrogates as reference is plotted in green. Correlation is significant where the data correlation (red) is higher than reference correlation (green). Note similarity especially in the intervals from ~38–30 ka (red arrows), and dissimilarity especially between ca. 15 and 30 ka. Note likely distortion of the loess signal at ~40-39 ka due to occurrence of a tephra layer related to the Campanian Ignimbrite (CI, red box).

A loess-palaeosol record from Rasova at the Lower Danube^12^ spanning the last 45 ka, which records millennial-scale climate variability in various proxy data including the frequency dependent magnetic susceptibility, is investigated regarding its (dis)similarity with a low-pass filtered Greenland signal. As for the comparison between Greenland δ^18^O and Sofular cave δ^13^C, the similarity is high for the time intervals from ~40–30 ka and ~15–0 ka, while less similar in the interval from ~30–15 ka. For Rasova, the occurrence of the Campanian Ignimbrite/Y-5 tephra dated at ~40 ka^107^, often found in the Lower Danube loess records as a chronostratigraphic marker^65^, complicates signal comparison around 40 ka because of its considerable thickness (see Fig. 6). Furthermore, the high magnetic susceptibility signal related to this tephra, and tailing for ca. 1 kyr towards younger ages, is therefore clearly not only an palaeoenvironmental signal.

## Discussion

### Technical aspects of smoothing and comparison

Smoothing of reference datasets, here in steps of 1 kyr, allows for a detailed comparison in several examples (Figs. 1, 2, 5, 6), while in some cases (Figs. 3, 4) longer steps such as 3 kyr are useful as well. Comparison is done in a qualitative way in this study. Here, the aim is obtaining a better understanding of millennial-scale climate variability in European terrestrial records, and the visual comparison in Figs. 2–6 is a powerful tool for this purpose.

In order to statistically test observed similarities, it is possible to calculate windowed correlation coefficients. However, this is only useful when no (or little) flexibility in the time domain is present, which is not the case for these examples. Often it is necessary to apply quantitative correlation analyses. In our opinion, the application of phase randomized surrogates^29,30,81,82^ is the most suitable way of quantifying significance, and we apply it here. We include selected overlapping moving window correlations with moving window widths equivalent to the cut-off frequency (see Figs. 3, 6) to support our findings. Note that both the window length and the data resolution are factors influencing correlation and significance tests. For technical details, see the methods section and the computer code in supplementary materials. See also^93^ for a critical discussion of correlating loess proxy datasets.

Edge effects of filters are not particularly prominent in the shown examples, but such effects can be seen e.g. at the old end of data displayed in Fig. 1, and at the young end of the filters in Fig. 2. In this case, filters suggest rather low values at the young end of the dataset, which is clearly not suggested by the dataset itself.

### Smoothing of palaeoenvironmental signals in terrestrial sedimentary records

It is a well-known fact that many sedimentary geoarchives record a smoothed representation of past climate variations e.g.^108^, and references therein. Reasons are multifold and include low deposition rates, chemical, physical and biological mixing processes e.g.^109–112^, as well as low sampling resolution. Note that while these processes will act on different time scales and possibly with different time-delay to climate forcing, the here discussed filtering process represents a simplified model of the sum of these. Aeolian sediments can be expected to preserve a record of climate forcing over a considerable time span. Soil formation including (physical and chemical) weathering processes will act on varying depths, depending on the (climate driven) intensity of processes, and are modulated by deposition rates of aeolian material. Various processes contribute to a signal smoothing in terrestrial environments, and although several possible processes can be named, the complex interplay of these can be expected to be site specific. In practice, rather smooth sedimentary datasets are sometimes considered indicative for continuous deposition. Also final accumulation (and preservation) rates are depending on the depositional environment. Strong variability in proxy data with depth may be interpreted as either different sediment sources or variable local environmental conditions. Gaps in the sedimentary record pose a challenge for all depth/time series analyses methods. Here we assume a quasi-continuous sedimentation on millennial time scale, which has been convinclingy demonstrated in several cases e.g.^4,39^. Where this is not the case, and erosion and/or colluvial sediments are present, care must be taken not to interpret resulting features as palaeoclimate patterns.

### Comparison to smoothed reference datasets improves interpretations of proxy records

We advocate considering smoothed signals as comparison targets for geoarchives with different temporal resolutions and discuss this approach in the context of comparing low-pass filters of e.g. the high-resolution Greenland and Sofular records to terrestrial loess deposits. We clearly do not recommend this approach to answer research questions of timing, synchronicity (lead and lags) and duration of millennial-scale climate variability when no additional data is available. We do introduce this approach as a useful way to assess chronological correlations and to obtain an overview of how smoothed studied records may look like. All here discussed datasets show different imprints of millennial-scale climate variability, which would have influenced all discussed proxy datasets, and may have imposed palaeoenvironmental (and therefore data) variability in the period band of 2–15 kyr. This period range in between orbital- and millennial time scales is often difficult to use for comparison (and correlation) of palaeoclimate proxies. This is due to the absence of reference datasets showing a clear variability in this period band.

Figure 4 shows selected low-pass filters for the Greenland δ^18^O data, the Imbrie and Imbrie (1980) ice model as parametrized by Lisiecki and Raymo (2005), and also the orbital parameters precession and obliquity^99^ for the last 130 ka. Differences in timing of the filter maxima representing the warmest phase of MIS 3 according to the discussed reference records are apparent and range from ~60 to ~52 ka. This makes an unambiguous and precise correlation challenging, where it is not clear which of these reference datasets is most suitable. Especially the similar pattern but different timing of Northern Hemisphere insolation and the Imbrie and Imbrie (1980) ice model allow for ambiguity. Several loess datasets record three clear intervals reflecting rather warm and relatively humid climatic conditions during MIS 3 e.g.^113^. Such a pattern is not found in insolation- or ice models, but it is observed in the smoothed Greenland data (Fig. 4, red arrows). A good relation between the soil complexes related to MIS 3 and their magnetic susceptibility signal and a smoothed Greenland δ^18^O signal can be achieved (Fig. 4), especially when low-pass filtering the original record with a cut-off period of (around) 9 kyr. Especially for MIS 3, the low-pass filters may therefore be useful correlation targets, particularly where the resolution of geoarchives is in between millennial and orbital time scales. In several time intervals (~40–30 ka, ~15-0 ka) loess data can be correlated more clearly to low-pass filters of Greenland isotope data than to other reference datasets (Fig. 4). This comparison allows therefore for an interpretation regarding the timing of the MIS 3 soil formation, and its dominant forcing.

A last glacial weathering proxy dataset from the Czech Republic in loess and loess-like sediments^106^ shows varying weathering intensity and changing sedimentation rates^106^ (Fig. 5). We compare this dataset to Greenland δ^18^O data and its smoothed representations. The comparison allows for assigning different resolutions of the acquired palaeoenvironmental signal, related to changes in sedimentation rate. In this case, a rather high recording resolution of ~1 ka around 60 kyr can be inferred, which decreases to ~2–3 kyr from ~55–40 kyr, and thereafter is in the order of ~5 kyr. This example demonstrates that a comparison to different low-pass filtered reference data allows an improved proxy data understanding, which may be used for time scale evaluation and construction.

As a third example, we use a loess profile from the Lower Danube^12^ (Fig. 6). Some maxima in the (frequency dependent) magnetic susceptibility around 40-30 ka are similar to the smoothed Greenland reference data and indicate a recording resolution of ~1 kyr. Thereafter, the similarity between Greenland reference data and the loess archive becomes less obvious until the beginning of the Holocene, which can be assessed visually or via correlation analysis. This, in combination with the comparison of Greenland and Sofular cave data, may suggest an interpretation that the Black Sea region was strongly linked to the Northern Hemisphere climate evolution (here approximated by the Greenland reference dataset) at least until ca. 30 ka, and thereafter the link to Greenland and Sofular cave may have been weaker. In this case, correlation is mostly significant above 95% confidence level until around 35 ka, and from ca. 25 ka towards present.

### Smoothed reference datasets as correlation templates, with focus on the last glacial cycle and MIS 3 in European loess

In addition to the use for an improved understanding of proxy records, the low-pass filtered reference datasets^1,54^ may be employed as correlation targets. The comparison of (terrestrial) proxy data (challenging to independently date) to smoothed reference datasets may allow assigning a time scale through correlation, ideally backed up by independent chronological data.

We show that correlation of palaeoclimate proxy data from MIS 3 is especially challenging when millennial-scale variability is not clearly identifiable in proxy-data and cannot be used to establish a reliable time scale (e.g. Figure 4). In such cases, independent dating may point to a match to precession, ice models or a smoothed Greenland signal. We show several cases^106,113,114^ where patterns are best explained by a smoothed Greenland signal, and considerable mismatches to all ‘classical’ correlation targets exist. The (palaeoenvironmental) interpretation of other loess datasets may benefit from similar comparisons to reference datasets between millennial and orbital scales.

The here presented examples come from terrestrial loess geoarchives covering the last glacial cycle. As expected, our analysis shows that the palaeoclimate proxy signal was not recorded with a constant temporal resolution. We find that some palaeoclimatic and/or sedimentary conditions can facilitate the recording of a clear palaeoenvironmental signal. This is to be kept in mind for correlations in general, and especially when interpreting weak signals with low variability.

## Conclusions

Here we discuss a new approach in defining low-pass filters with changing cut-off frequencies from well dated high-resolution reference records of millennial-scale climate variability for the last glacial cycle^1,54^. The results can exemplary explain proxy datasets with less established time control better than the use of commonly applied high-resolution reference datasets. This improved correlation is proposed to allow for a better understanding of proxy datasets, and possibly more reliable correlative age models, which are especially powerful when supported by independent and reliable dating.

We propose using low-pass filtered reference records when palaeoclimate datasets show variability between millennial and orbital scales, and no other suitable reference datasets are available. Applying this approach, we

(a) explain observed patterns and their relation to millennial-scale climate variability in several terrestrial examples,

(b) can gain insight in the temporal resolution of geoarchives, and

(c) propose these filtered/smoothed signals as correlation targets for records lacking high frequency (millennial-scale) recording but showing smoothed climate variability on supra-millennial-scales (~2–15 kyr).

Moreover, low-pass filters as a simplified model improve our understanding how low sedimentation, bioturbation, and pedogenesis may result in a smoothing of terrestrial (loess) records.

In our opinion, comparing smoothed records to reference data may be a step forward especially for last glacial stratigraphies, where millennial-scale patterns may be expected, but are not directly recorded in full resolution in some (marine, lacustrine, terrestrial) geoarchives.

## Methods

### Filter settings

Filtering is done here using a Taner filter as implemented in the ‘astrochron’ R package^29–31^. More details on Taner filters are available in^115^ and supplementary materials. We use several cut-off frequencies in 1- kyr steps, from 1 to 15. The roll-off rate is set to 10^20^ for all filters; it determines the spectral filter response. An exemplary sensitivity analysis of the roll-off rate is provided in the supplementary materials. Different settings of the roll-off rate are expected to lead to similar results and can be tested using the appended R scripts. Note that we use a single filtering step for processes acting on different time scales. Although we consider this an improvement it is a simplification at the same time.

### Reference datasets and European terrestrial context

Here, we low-pass filter several high-resolution reference records on their individual age models, which show high-frequency oscillations on millennial time scales (Figs. 2, 3). We use the Greenland δ^18^O data^54,55^ on the AICC2012^55,56^ time scale, and the δ^13^C datasets from Sofular cave^1^ in the Black Sea area. Greenland δ^18^O is commonly regarded as a proxy dataset for past temperature variability over Greenland, possibly representative for the North Atlantic or northern hemisphere temperature evolution^54^. However, it also represents the most commonly used palaeoclimate and chronological reference dataset for disentangling past climate variability during the last glacial cycle in the Northern Hemisphere^2,3,12,13,39,116–120^. The Sofular cave δ^13^C dataset^1^ represents a reliably and independently U/Th dated terrestrial cave record that would allow for direct comparison with (south-eastern) European loess deposits. This is because the δ^13^C isotopic composition of cave calcite is climate-driven by hydroclimatic processes directly affecting the soil and vegetation composition above the cave^1^ and thus provide a direct analogue to biogeochemical processes affecting loess records.

Taner low-pass filters are used as correlation target and compared to several geoscientific datasets: (a) Two rock magnetic loess records covering the last glacial cycle from Mošorin/Serbia in the Carpathian Basin^113^, and the magnetic susceptibility stack from the Loess Plateau in China^114^ (Fig. 4); (b) a weathering proxy dataset from partly pedogenetically overprinted loess at Bíňa/Slovakia^106^ (Fig. 5); and (c) a rock magnetic dataset from Rasova/Romania in the Lower Danube Basin^12,121^ (Fig. 6). These datasets have been chosen for several reasons. The datasets of magnetic susceptibility from loess (Serbian dataset and Chinese loess stack) spanning the last glacial cycle^113,114^ are investigated to demonstrate issues in comparing loess data to insolation data^99^ and the ice model by Imbrie and Imbrie (1980). These datasets demonstrate the usefulness of smoothed millennial-scale records in interpreting the patterns found in terrestrial geoarchives. The Rb/K weathering record^106^ is used here for demonstrating the potential of obtaining a better understanding of loess (like) records as palaeoclimate archives on millennial-scale by comparison to both a high-resolution proxy record (here: the Greenland isotope record), and also its smoothed representations following different cut-off frequencies. Finally, the datasets from the loess record from Rasova in the Lower Danube Basin on its individual time scale established by a combination of luminescence dating, tephrochronology and correlation^12^ is used to show how avoiding noisy high-frequency components can help interpreting these records in respect to both hemispheric-scale and/or more local palaeoclimate influences. The code of computations associated with this manuscript is available in supplementary materials.

Classical and often used reference datasets in palaeoclimatic comparisons and (chronological) correlations include Northern Hemisphere summer insolation^99^, and the Imbrie and Imbrie (1980)^108^ ice model as parametrized for an oxygen isotope stack^98^. The (frequency dependent) magnetic susceptibility from loess-palaeosol records is commonly used as proxy for inferring the degree of pedogenetic overprinting of aeolian material, which is largely controlled by temperature and especially moisture availability^104^. Proxy data from the Chinese Loess Plateau and European loess belt^101–105^ show that loess magnetic susceptibility is a reliable proxy for inferring palaeoenvironmental conditions and associated palaeoenvironmental change through time.

### Correlation analysis

Correlation can be computed through the calculation of correlation coefficients for linear and nonlinear systems e.g.^122,123^, here we use the Spearman rank correlation coefficient for quantification of similarity. Using classical statistics to calculate the significance of the correlation is not plausible here since the data are serially correlated. In our opinion, the application of phase randomized surrogates^29,30,81,82^ is the most suitable way of quantifying significance. This method is applied as implemented in the ‘astrochron’ R package^29,30^. Here, we compare correlation coefficients to moving window significant levels of correlation (Figs. 3, 6). For more details, please see the computer code in supplementary materials.

## Supplementary information

Supplementary information.

Supplementary information 2.
