# Supplementary material for: Smoothed millennial-scale palaeoclimatic reference data as unconventional comparison targets: Application to European loess records
Source: Sci Rep. 2020 Mar 25;10:5455. doi: 10.1038/s41598-020-61528-8 (PMC7096450; doi:10.1038/s41598-020-61528-8)
Supplement: Supplementary file 2 — Supplementary information 2. [file 41598_2020_61528_MOESM2_ESM.pdf]

Supplementary Materials to the manuscript ‚Smoothed millennial-scale paleoclimatic reference data as unconventional comparison and correlation targets: Application to last glacial cycle European terrestrial records'

Christian Zeeden[1,2*], Igor Obreht[3], Daniel Veres[4], Stefanie Kaboth-Bahr[5,6], Jan Hošek[7, 8], Slobodan B. Marković[9], Janina Bösken[10], Frank Lehmkuhl[10], Christian Rolf[1], Ulrich Hambach[11]

[1] LIAG, Leibniz Institute for Applied Geophysics, Hannover, Germany

[2] IMCCE, Observatoire de Paris, PSL Research University, CNRS, Sorbonne Universités, UPMC Univ. Paris 06, Univ. Lille, Paris, France

[3] Organic Geochemistry Group, MARUM-Center for Marine Environmental Sciences and Department of Geosciences, University of Bremen, Bremen, Germany

[4] Romanian Academy, Institute of Speleology, Cluj-Napoca, Romania

[5] Institute of Earth Sciences, Ruprecht-Karls-Universität Heidelberg, Heidelberg, Germany

[6] Institut für Geowissenschaften, Universität Potsdam, Potsdam, Germany

[7] Czech Geological Survey, Prague, Czech Republic

[8] Center for Theoretical Study, Charles University and the Academy of Sciences, Czech Republic

[9] Chair of Physical Geography, Faculty of Sciences, University of Novi Sad, Novi Sad, Serbia

[10] Department of Geography, RWTH Aachen University, Germany

[11] BayCEER & Chair of Geomorphology, University of Bayreuth, Germany

*Correspondence to Christian Zeeden, christian.zeeden@leibniz-liag.de

Supplementary Materials contain:

This document (Supplementary_Materials.docx), containing a documentation of a sensitivity analysis of low-pass filters and their dependency on roll-off rate.

And a compressed .zip file including:

An excel document (Supplementary_data.xlsx) containing:

- The autoregressive noise as in Fig. 1 (tabs: Fig1a, Fig1b)
- Low-pass filters of Greenland δ^{18}O data (tab: Greelnland_d18O) as in Figs. 2, 3
- Low-pass filters of Sofular cave δ^{13}C data (tab: Sofular_d13C) as in Fig. 3

Following R scripts:

Fig1.R: Calculations and raw plot of Fig.1. Please note that the auto-regressive noise generation is not reproducible, but the data from the realisation in this manuscript are available in the supplementary excel sheet.

Fig2.R: Calculations and raw plot of Fig.2

Fig3.R: Calculations and raw plot of Fig.3

Fig6.R: Calculations for Fig.6, including plotting of results and data export. Note that the plot in the manuscript was not directly created in R, therefore the final plot is not included in the R script.

Supplements_test_rollrate.R: Documents the sensitivity analysis of low-pass filters dependency on the roll-off rate

In total Supplementary Information contain seven documents, and two files.

Sensitivity analysis of low-pass Taner filters and their dependency on roll-off rate

Here three different roll-off rates for Taner filters (Taner, 1992) are compared. Taner filters are used as implemented in the R 'astrochron' package (Meyers, 2014; R Core Team, 2017). The roll-off rate describes the sharpness of the implemented cut-off frequency, with low roll-off rates representing soft cut-off frequencies (top right Fig. S1), and high roll-off rates implement sharp cut-off frequencies (top right Figs. S2, S3). Often rather high roll-off rates in the order of 10^3 to 10^50 are applied to make a clear separation between investigated signal, and not investigated signal (Zeeden et al., 2018). In practice, usually roll-off rated from ca. 10^5 and higher give similar results. See Fig. S4 for the limited effect here.

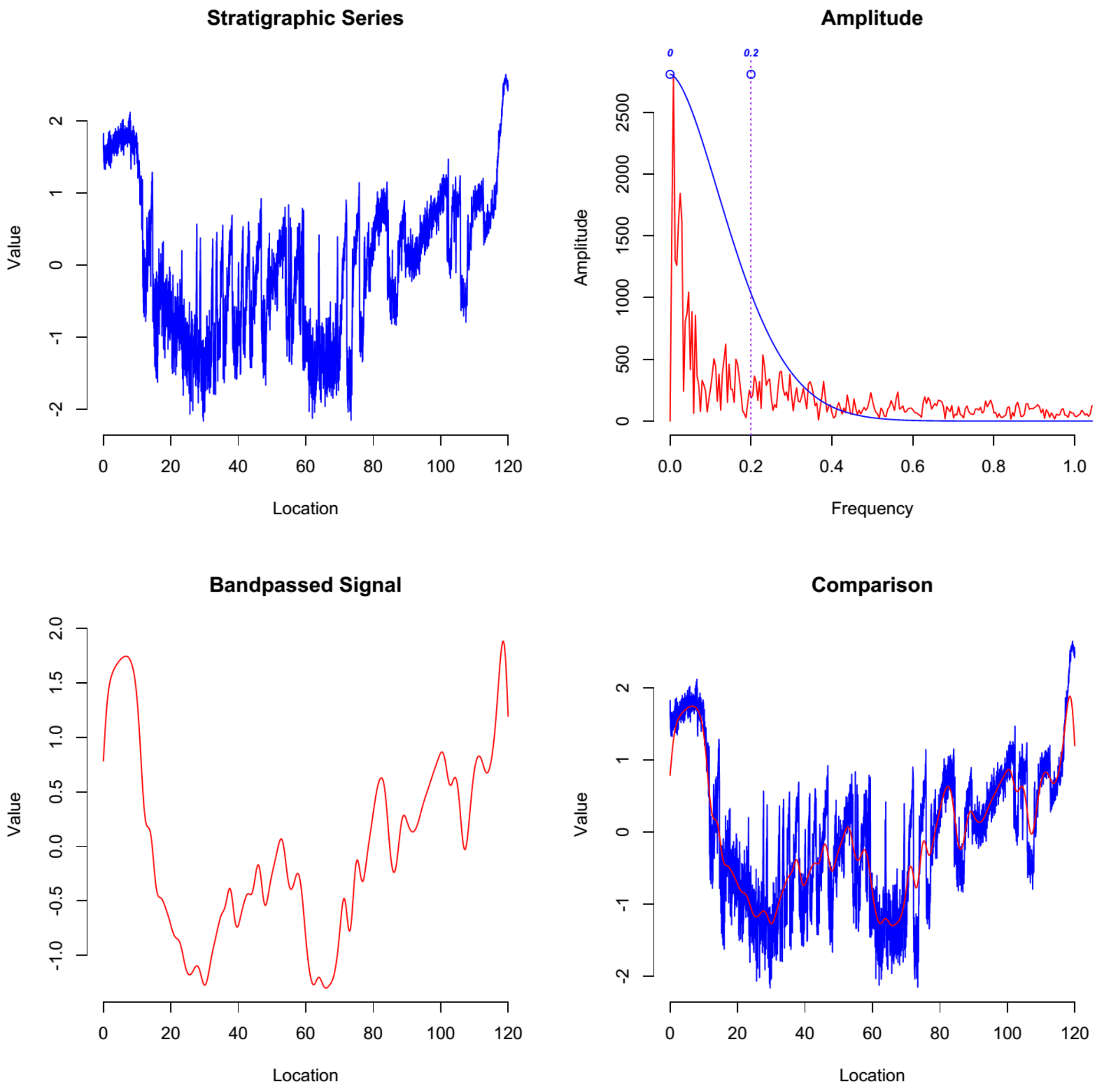

Figure S1: Results of low-pass filtering the Greenland $\delta^{18}O$ (North Greenland Ice Core Project Members, 2004) record using a cut-off frequency of 1/5 (1/ka), and a very low roll-off rate of 5. The top left panel shows the detrended and standardized record, the top right panel shows a spectrum and the Taner filter response (blue) with the cut-off frequency of 1/5=0.2 (1/ka). The bottom left

panel shows the resulting low-pass filter, and the bottom right panel compares signal (blue) and filter (red).

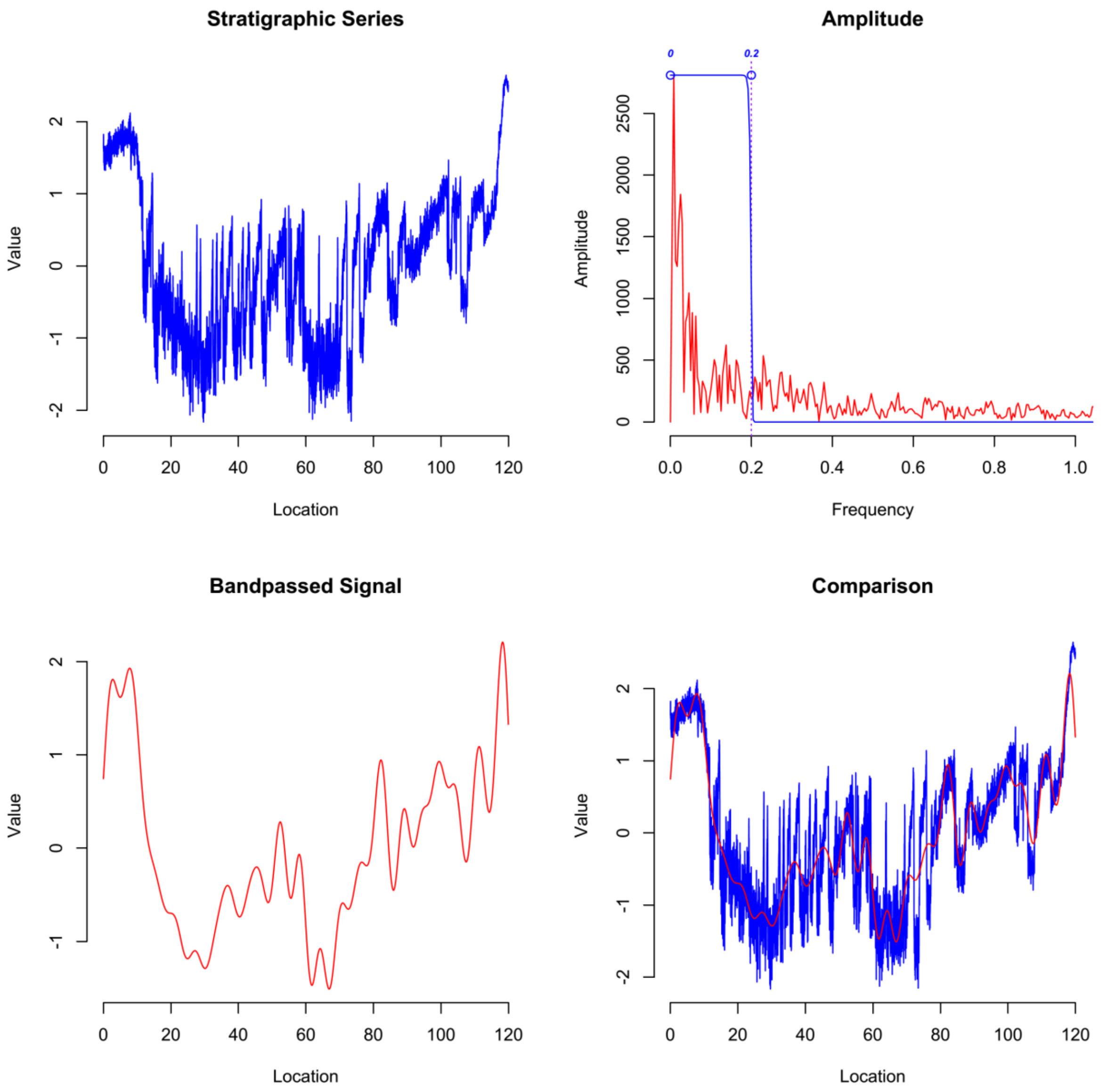

Figure S2: Results of low-pass filtering the Greenland δ^{18}O record using a cut-off frequency of 1/5 (1/ka), and a roll-off rate of 10^20. See also heading of Fig. S1.

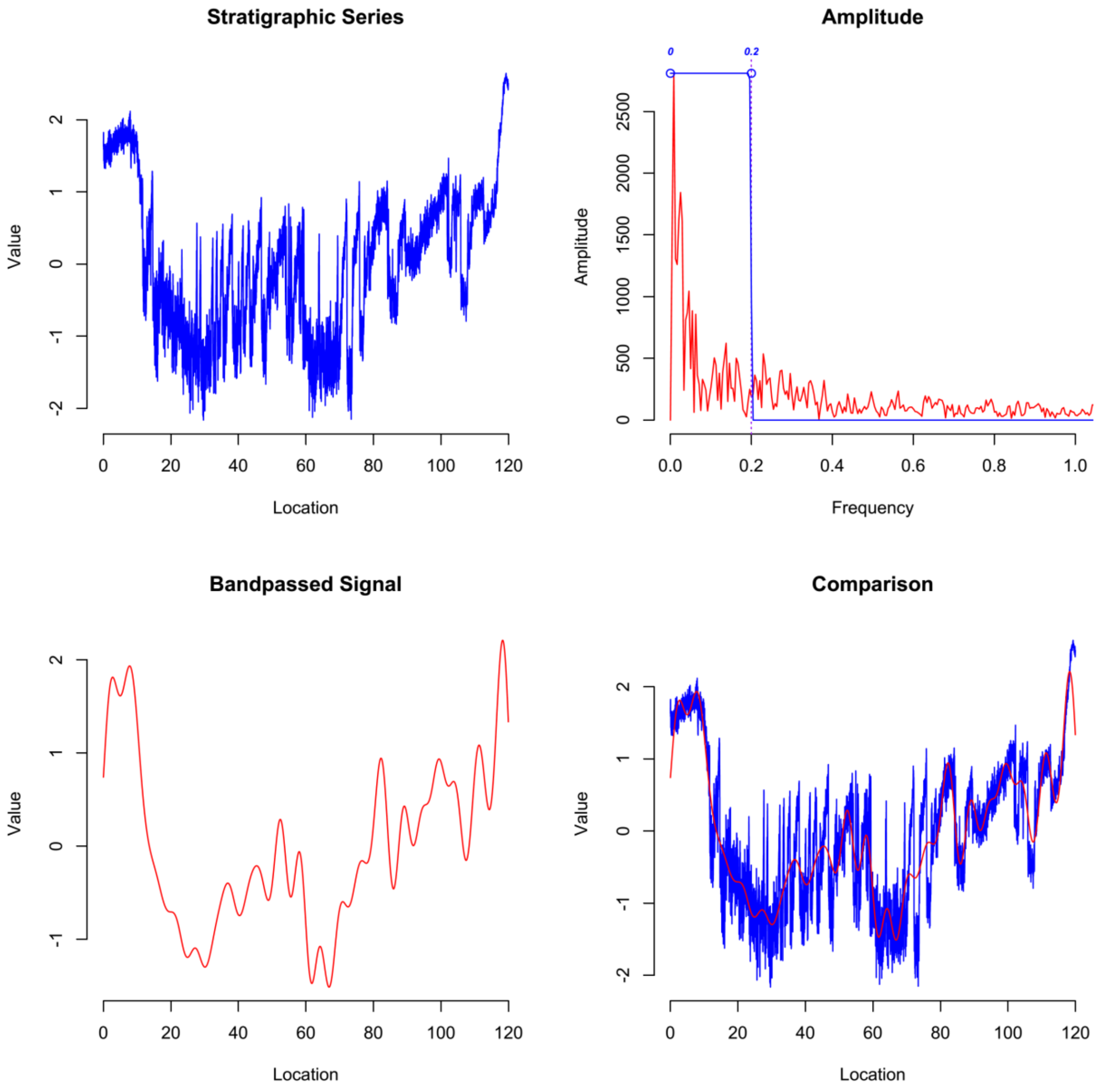

Figure S3: Results of low-pass filtering the Greenland $\delta^{18}O$ record using a cut-off frequency of 1/5 (1/ka), and a roll-off rate of 10^50. See also heading of Fig. S1.

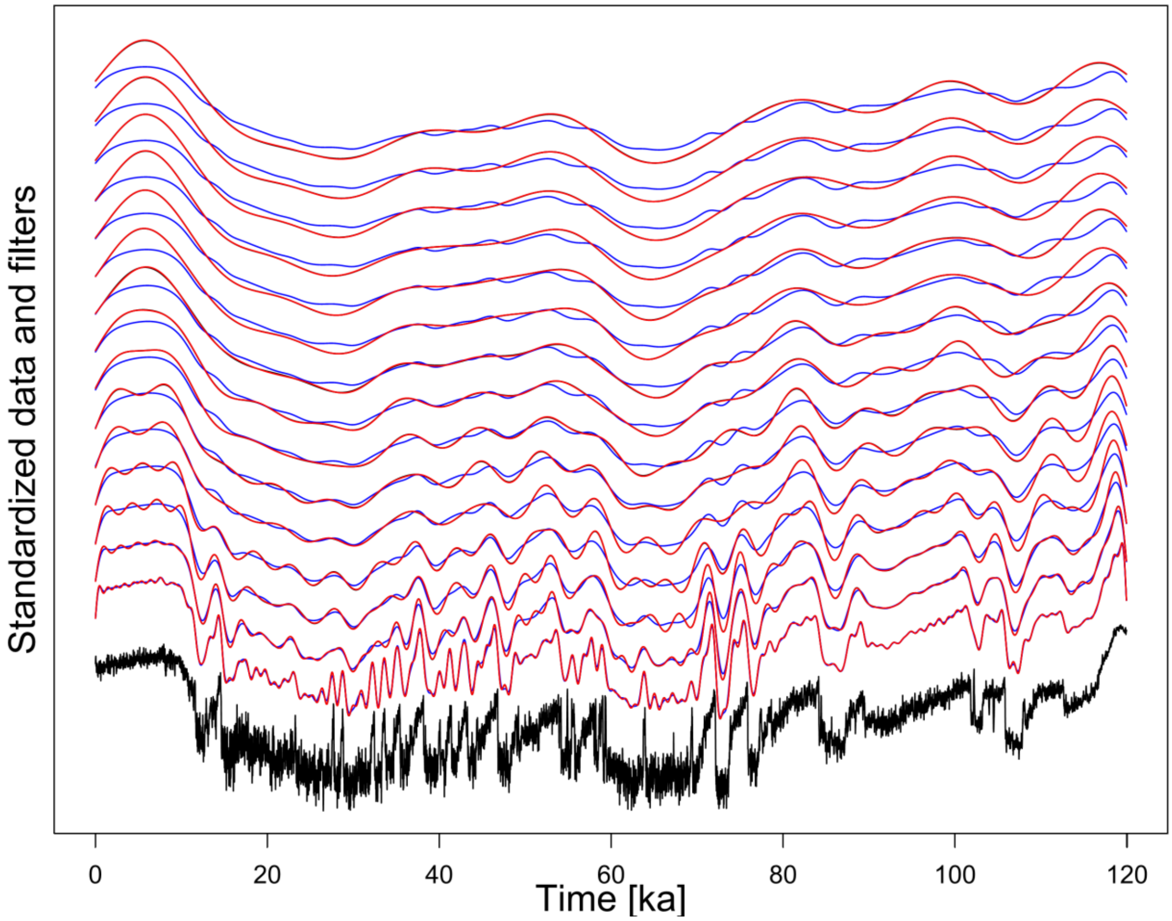

Figure S4: Comparison of Greenland $\delta^{18}O$ record (North Greenland Ice Core Project Members, 2004) low pass filters, using cut-off frequencies of 5 (1/ka; blue), 10^20 (black), and 10^50 (red). Note that the filters with higher roll-off rates (10^20, 10^50) are (almost) indistinguishable in this figure here.

References used in Supplementary Materials:

Meyers, S.R., 2014. astrochron: An R Package for Astrochronology Version 0.8.

North Greenland Ice Core Project Members, 2004. High-resolution record of Northern Hemisphere climate extending into the last interglacial period. Nature 431, 147–151. https://doi.org/10.1038/nature02805

R Core Team, 2017. R: A Language and Environment for Statistical Computing.

Taner, M.T., 1992. in: Attributes revisted (Technical Report, Rock Solid Images, Inc), url: http://www.rocksolidimages.com/attributes-revisited/#_Toc328470897.

Zeeden, C., Kaboth, S., Hilgen, F.J., Laskar, J., 2018. Taner filter settings and automatic correlation optimisation for cyclostratigraphic studies. Comput. Geosci. 119, 18–28. https://doi.org/10.1016/j.cageo.2018.06.005
